# If you build it, will they come? Social, economic, and psychological determinants of COVID-19 testing decisions

**DOI:** 10.1371/journal.pone.0252658

**Published:** 2021-07-14

**Authors:** Brea L. Perry, Brian Aronson, Ashley F. Railey, Christina Ludema

**Affiliations:** 1 Department of Sociology, College of Arts and Sciences, Indiana University, Bloomington, IN, United States of America; 2 Department of Epidemiology and Biostatistics, School of Public Health, Indiana University, Bloomington, IN, United States of America; University of Utah, UNITED STATES

## Abstract

**Background:**

The efficacy of testing and tracing programs to reduce COVID-19 transmission hinges not only on widespread access to testing, but also on the public’s willingness to participate in them. To the extent that testing intentions are patterned by social determinants of health, this constitutes an understudied mechanism of disparities in COVID-19 morbidity and mortality.

**Design:**

Using data from a representative household probability sample, the Person to Person Health Interview Study (n = 935), sociodemographic, economic, and psychological determinants of testing considerations were evaluated across six domains: treatment affordability, ability to work if positive, hospital effectiveness, symptom severity, proximity to infected, and risk of transmitting to others.

**Results:**

Findings demonstrated significant differences in testing motivations across race/ethnicity, education level, socioeconomic status, and worry about self and loved ones. Notably, Black (p<0.01) and Latino (p<0.05) respondents and those experiencing financial strain (p<0.001) were disproportionately likely to indicate that resource factors would influence their decision to get tested. Desire to reduce transmission and concern about proximity to the infected were reported among those who expressed COVID-19 worries (p<0.001).

**Conclusion:**

Public health efforts to combat the COVID-19 pandemic must address social, economic, and psychological factors that enable and constrain individual behavior. Increasing access to preventative interventions and technologies, including vaccines, is unlikely to markedly reduce morbidity and mortality without effective messaging and economic support to improve uptake in vulnerable populations.

## Introduction

Strict COVID-19 social distancing through bans on mass gatherings, closing schools, and working remotely have been effective worldwide in reducing the spread of the virus, but are not a feasible long-term solution given the enormous cost to social and economic wellbeing. This has prompted research on “exit strategies” that would permit activities and economies to remain open while still protecting healthcare resources and reducing viral transmission. Epidemiologists have advocated for robust testing and contact tracing as a potential solution to balance public health and economic priorities, with research suggesting that an optimal system could prevent up to 80% of all transmission [1]. However, the success of testing and tracing efforts hinges on minimizing the delay between symptom onset and testing and isolation of index cases [2] with a gap of 48 hours or less being critical for reducing the reproductive number [1]. Developing reliable models to guide such strategies thus requires information on social and behavioral heterogeneities that impact transmission and may influence willingness to test [3].

Though willingness of people experiencing symptoms of COVID-19 to participate in testing and tracing programs is likely to be an essential determinant of their efficacy, few studies have examined factors contributing to testing intentions [4, 5]. Initially, healthcare providers served as gatekeepers, but as tests have become more readily available, patient self-selection is becoming increasingly important. In the current pandemic, we have seen numerous examples of widespread testing refusal, and up to 30% of people report that they would not take a free COVID-19 test if it were offered to them [5, 6]. Emerging evidence suggests that perceptions of infection risk [7, 8], desire to avoid infecting others [5], and anticipated financial costs associated with testing, treatment, and quarantining may affect willingness to be tested for COVID-19 [4, 9]. However, to date, there have been no comprehensive, representative studies of motivations affecting COVID-19 testing intentions.

This knowledge gap regarding testing intentions is significant because unwillingness to be tested is likely to vary systematically across social determinants of health. Specifically, the ability to effectively respond to test results likely varies by race/ethnicity, immigration status, socioeconomic status (SES), and other factors correlated with insurance status, ability to pay for treatment, and access to well-resourced hospitals [10, 11]. In other words, knowledge does not always bring power. Likewise, these same social determinants are associated with the presence of competing demands, including disproportionate potential for job loss, inability to work from home, and inability to absorb income shocks associated with quarantine [12–14]. For individuals in precarious economic positions, knowing one’s COVID-19 status may result in income shocks.

Group differences in testing intentions due to systemic inequalities have the potential to exacerbate economic and health disparities in the US. Transmission models that do not account for testing intentions and behavior are likely to poorly estimate the efficacy of testing and tracing strategies. Moreover, understanding motivations for testing intentions across different social status groups is essential for developing effective and equitable strategies to prevent transmission, hospitalization, and mortality. In the current paper, we use data from one wave of a state representative panel study of Indiana residents to examine the influence of sociodemographic, economic, and psychological determinants on six motivations for testing: treatment affordability, ability to work if positive, hospital effectiveness, symptoms severity, proximity to infected, and risk of transmitting to others. The results of our study intend to inform the design and dissemination of testing strategies, as well as highlight social inequalities in testing motivations that may influence the reliability of transmission reducing models.

## Materials and methods

This research was approved by the Indiana University Institutional Review Board. Written informed consent was obtained. To understand which factors influence COVID-19 testing considerations, we use data from the COVID-19 module of the Person to Person Health Interview Study (P2P). The P2P module contains detailed information about the demographics, health, economic status, and COVID-19 experiences of 1,026 households across Indiana to assess the initial impacts of the virus. Inclusion depended upon participation in previous data collection of the P2P, which used a household-based stratified probability sampling method to collect data on a representative sample of residents across the state of Indiana. Respondents participated in interviewer-administered surveys at two points in time, in the year prior to the pandemic (via face-to-face interview) and in the early stages of COVID-19 (via phone interview; between 2020-04-03 and 2020-05-30). We include in our analysis those respondents from the COVID-19 module who answered all questions in our models (n = 935) and we adjust estimates with post-stratification weights to match populations values in Indiana (See S1 Appendix in S1 File for additional information about sampling methods and variable weighting). Missing values occurred for no more than 5% of any one variable.

### Measures

Our outcomes assess whether respondents’ decisions about whether or not to be tested for COVID-19 would be affected by one of the following six factors: The affordability of treatment if the respondent tested positive (*treatment affordability*), whether testing positive would require respondents to stop working (*ability to work if positive*), whether “hospitals have what they need to treat” individuals who test positive for COVID-19 (*hospital effectiveness*), the severity of respondents’ symptoms (*symptom severity*), whether respondents had been around someone who had tested positive for COVID-19 (*proximity to infected*), and the risk that respondents could infect other people (*risk to transmit to other*), with response options 0 = strongly disagree, 1 = disagree, 2 = agree, and 3 = strongly agree.

We choose predictors that can help us identify how demographic, economic, and psychological factors influence COVID-19 testing considerations. Among our demographic measures, we include four exclusive/nonintersecting race/ethnicity variables (non-Latino *White*, non-Latino *Black*, *Latino*, and *other*), four dichotomous measures of educational attainment (*bachelors or higher*, *some college*, *high school*, and *less than high school*), three dichotomous age variables (*age 15–34*, *age 35–54*, *age 55+*; in years), and one dichotomous sex variable (*female*). We examine economic factors that contribute to testing decisions via respondent’s insurance status (*uninsured*), and with a *financial strain index* (Cronbach’s alpha = 0.76; standardized) based on each respondent’s agreement to questions regarding concerns for enough money to buy food, general financial security, and housing insecurity or whether the respondent will have a place to live. Finally, we measure how psychological factors contribute to COVID-19 testing considerations with three indicators: (1) A *COVID worry index* (Cronbach’s alpha = 0.84; standardized) based on the extent that respondents agreed that they worry about catching COVID-19, worry about getting severely ill from COVID-19, worry about COVID-19 prevention, worry about friends and family catching COVID-19, and worry about friends and family becoming seriously ill from COVID-19 (See S2 Appendix in S1 File for results from exploratory factor analysis); (2) A dichotomous variable tracking whether respondents have had any *COVID symptoms*; (3) A dichotomous measure of *knowing someone with COVID-19* indicates whether the respondents knows someone personally, regardless of relationship, who has contracted the virus. We also estimated models with indicators for health (physical health, diabetes, smoking behavior), but these were excluded from our final analyses due to their poor predictive power.

### Statistical analyses

First, we used bivariate statistics to estimate the association of each outcome with race/ethnicity, education, age, financial strain, and COVID-19 worry. Next, we used a series of ordinal logistic regression models to estimate how multiple factors work in tandem to influence COVID-19 testing considerations. All models included the same sample and set of predictors. We also adjusted each model with post-stratification weights and clustered all standard errors by shared county. Results are presented graphically in the main paper, with full regression results provided in S3 Appendix in S1 File. Graphical tests of the proportional odds assumption for ordinal outcomes are presented in S4 Appendix in S1 File.

## Results

### Descriptive statistics

Table 1 provides univariate descriptive statistics for all variables. Overall, the demographic characteristics of the weighted P2P sample align with those of Indiana residents [15]. Half of respondents identified as female, 84% identifying as White, and mean age was 53.2 years. Rates of agreement with each outcome varied widely, with 21% of respondents agreeing that their decision to test for COVID-19 would be influenced by their ability to work if tested positive and a majority (83%) agreeing that their proximity to people infected with COVID-19 would influence their decision to get tested. In general, respondents were less likely to agree that resource considerations (treatment affordability, ability to work if positive, and whether their hospital is adequately resourced) would influence them relative to other types of motivations.

**Table 1:** Descriptive statistics.

Variable	n (%)	Mean (SD)	Min	Max
Dependent				
*Treatment Affordability*	342 (34%)		0	1
*Ability to Work if Positive*	211 (21%)		0	1
*Hospital Effectiveness*	477 (47%)		0	1
*Symptom Severity*	785 (78%)		0	1
*Proximity to Infected*	846 (83%)		0	1
*Risk to Transmit to Others*	721 (71%)		0	1
Age		53.2 (18.2)	19	100
Female	630 (61%)		0	1
Race/ethnicity				
*Non-Latino Black*	89 (9%)		0	1
*Non-Latino White*	865 (84%)		0	1
*Other*	33 (3%)		0	1
*Latino*	39 (4%)		0	1
Education				
*Less than High School*	70 (7%)		0	1
*High School*	244 (24%)		0	1
*Some College*	395 (39%)		0	1
*Bachelors*	317 (31%)		0	1
Economic Factors				
*Uninsured*	62 (6%)		0	1
*Financial Strain Index*	1,026 (100%)	0 (1)	-2.95	1.97
Psychological Factors				
*COVID Worry Index*	1,025 (99%)	0 (1)	-2.03	3.19
*Has COVID Symptoms*	159 (16%)		0	1
*Knows someone w/ COVID*	348 (34%)		0	1

Note: Full sample includes all individuals without missing data (n = 1,026; missing = 91). Samples means and standard deviations on full sample. All dependent variables dichotomized into 1 = agreed or strongly agreed and 0 = disagreed or strongly disagreed. SD = standard deviation. % = percent of respondents in each category.

### Bivariate associations between social demographics, psychological factors, and COVID-19 testing considerations

**Fig 1:**
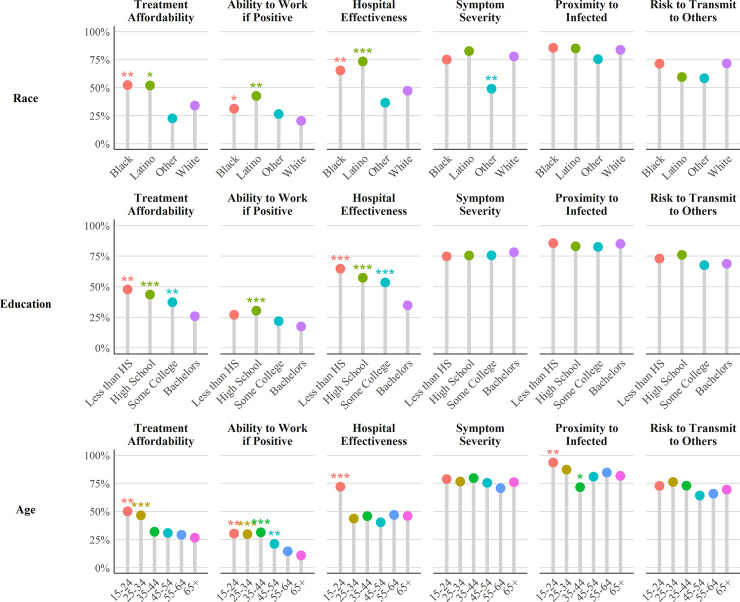
Factors associated with anticipated testing for COVID-19 by race, education, and age.

Fig 1 depicts the percentage of respondents from each category of race/ethnicity, education, and age that reported agreement or strong agreement to each motivation for COVID-19 testing (See S5 Appendix in S1 File for confidence intervals). There were significant demographic disparities in how resource considerations affect COVID-19 testing decisions. People who identify as Black or Latino, with less than high school education, and below age 35 reported substantially higher levels of agreement that treatment affordability, ability to work if positive, and hospital effectiveness would influence their treatment decisions than did other racial/ethnic, educational, and age groups. In other words, COVID-19 testing considerations among people in these demographic groups were disproportionately influenced by resource considerations. The remaining outcomes (symptom severity, proximity to infected, and risk to transmit to others) were rarely associated with race/ethnicity, education, and age. However, people of other racial backgrounds had lower agreement that the severity of COVID-19 symptoms would affect their decision to get tested for COVID-19.

**Fig 2:**
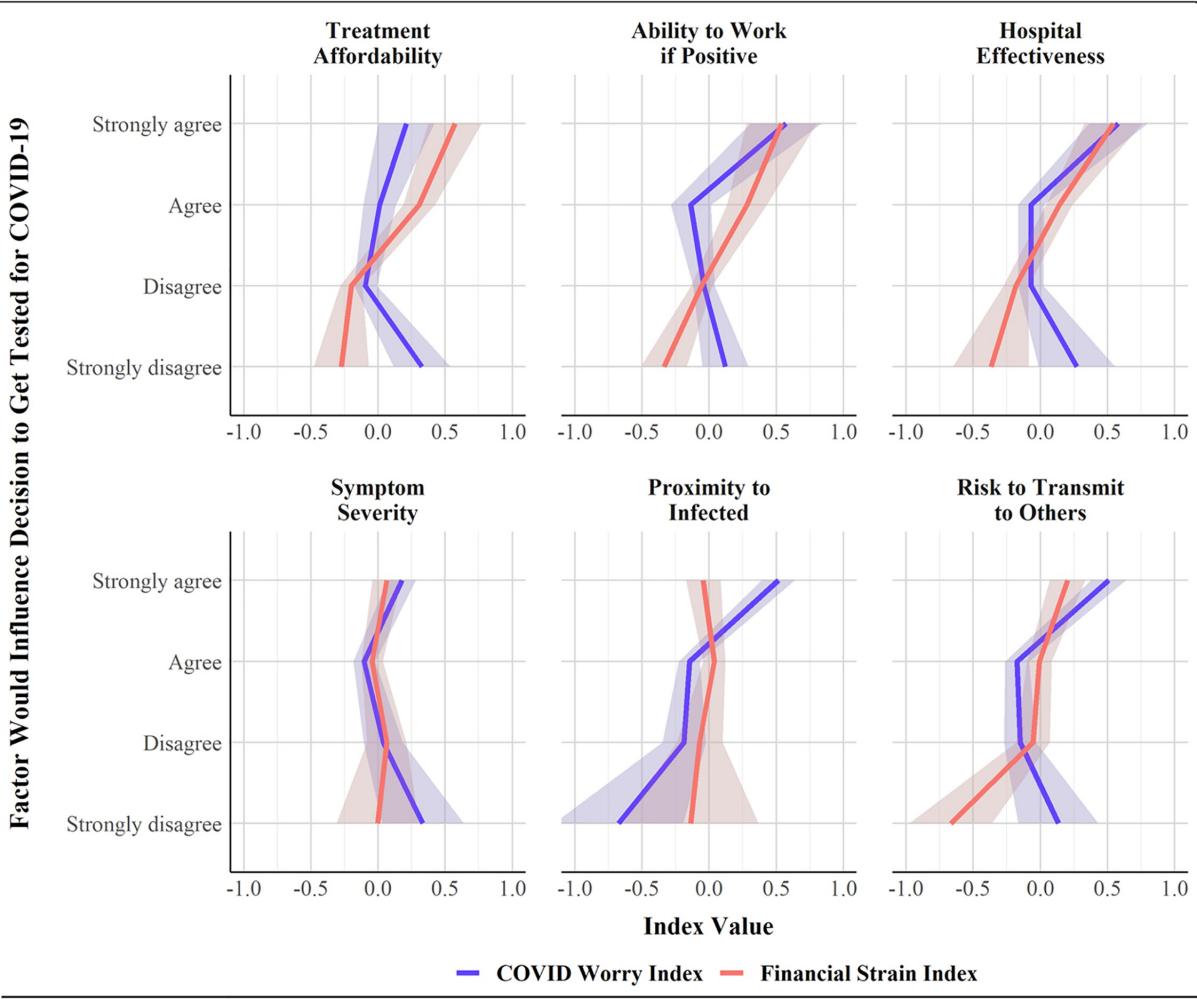
Association of COVID worries and financial strain with COVID-19 testing intentions.

We examined associations between economic precarity and testing motivations directly in Fig 2, which displays survey-weighted LOESS curves for each outcome by the financial strain index. Consistent with our demographic findings, we observed a linear association between financial worry and each resource consideration. For example, the average financial strain index among people who strongly disagreed that treatment affordability would influence their decision to get tested for COVID-19 was -0.27 standard deviations below the mean, whereas individuals who strongly agreed had an average financial strain index of 0.57 standard deviations above the mean. Fig 2 also depicts results for the COVID-19 worry index. This index had a modest positive association with most outcomes; COVID-19 worries only had a clear positive association with testing considerations for questions pertaining to respondent’s proximity to individuals who tested positive with COVID-19.

### Results from multivariate models predicting COVID-19 testing considerations

**Fig 3:**
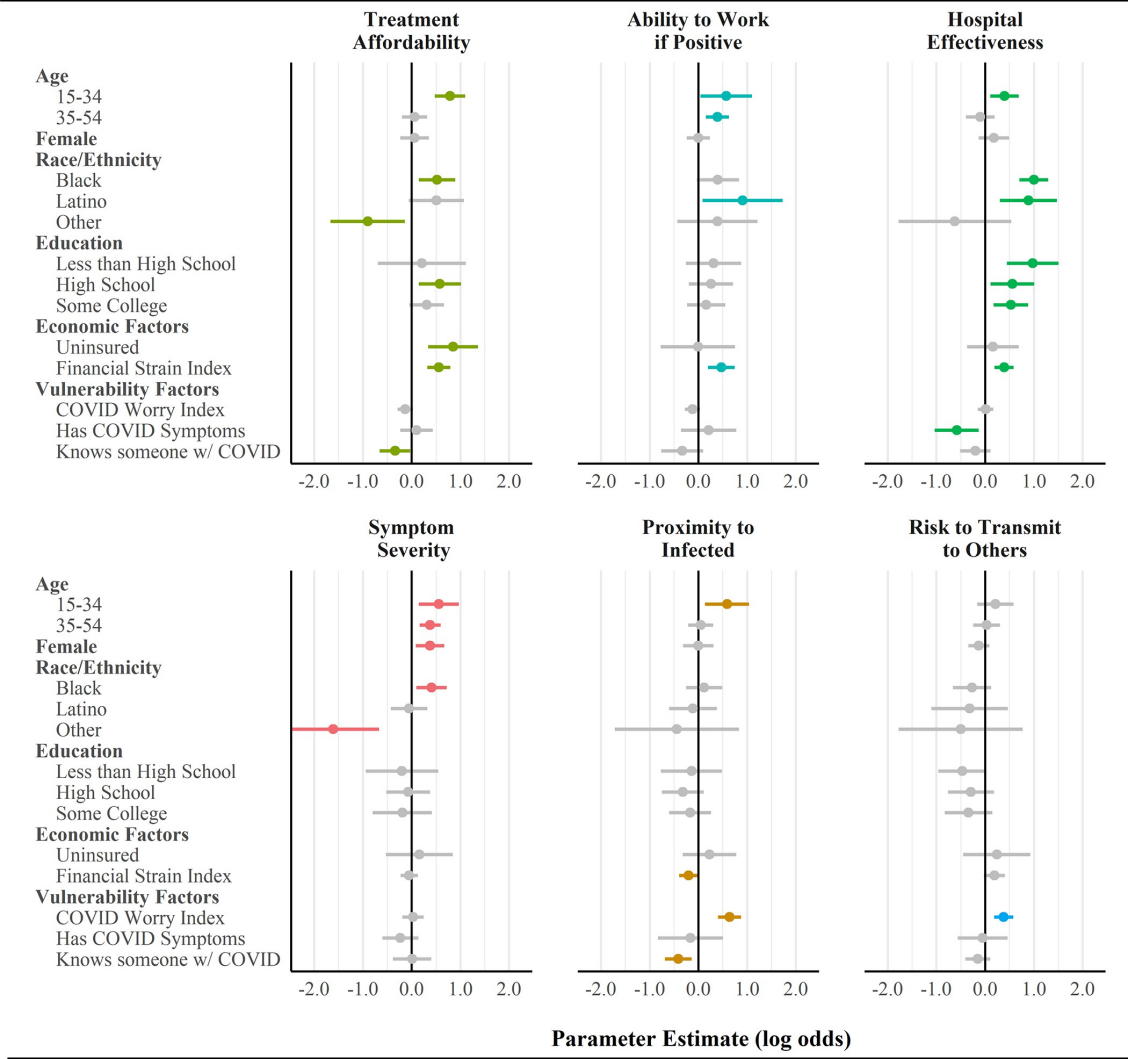
Multivariate regression results for the influence of sociodemographic, economic, and psychological determinants on testing intentions.

Fig 3 displays parameter estimates from ordinal logistic regression models predicting agreement that different criteria would affect respondents’ decisions to get tested for COVID-19. Full tables of results are reported in S1 File. Parameter estimates in Fig 3 are reported in log odds, with estimates above zero indicating factors that increased the odds of agreement to each outcome and estimates below zero indicating factors that were negatively associated with agreement. Estimates that were not statistically significant are colored grey.

Results from multivariate models presented in Fig 3 are largely consistent with bivariate findings reported in Fig 1. Compared to White respondents, Black and Latino respondents had significantly higher log odds of agreeing that their testing decisions would be influenced by the ability of hospitals to effectively treat COVID-19 symptoms (*b* = 1.00 and 0.89). Black respondents were also more likely to agree that the affordability of treatment would influence their decision to get tested for COVID-19 (*b* = 0.52), and Latino respondents were more likely to agree their testing decisions would be influenced by their ability to work if tested positive (*b* = 0.91). On the other hand, people of other racial groups were significantly less likely to report that treatment affordability (*b* = -0.90) or severity of symptoms (*b* = -1.61) would influence their decision to get tested for COVID-19. In comparison to people with a bachelor’s degree, individuals with less than a bachelor’s education were significantly more likely to state that treatment affordability and hospital effectiveness would influence their COVID-19 testing decisions. Compared to people aged 55+, younger people reported significantly higher agreement that considerations of all types (except risk to transmit to others) would influence their decision to be tested. Sex had a relatively limited association with treatment decisions; women only differed from men in their propensity to report the severity of their symptoms (*b* = 0.38) as an important consideration in their decision to get treated for COVID-19.

Economic and psychological factors also had significant associations with treatment considerations. Respondents who were uninsured were more likely to agree that treatment affordability would influence their decision to get tested for COVID-19 (*b* = 0.85). Experiencing financial strain was strongly associated with agreement that treatment affordability (*b* = 0.56), ability to work if positive (*b* = 0.47), hospital effectiveness (*b* = 0.39) would influence respondents’ decisions to get tested for COVID-19. Psychological factors often had an inverse association with outcomes that were highly influenced by resource factors. For example, knowing someone that tested positive for COVID-19 was negatively associated with the impact of treatment affordability on testing considerations (*b* = -0.34), and experiencing COVID-19 symptoms oneself was negatively associated with the impact of hospital effectiveness on testing considerations (*b* = -0.58). However, the COVID worry index had a large positive association with the extent to which recent proximity to infected individuals (*b* = 0.64) and one’s risk to transmit COVID-19 (*b* = 0.38) to other individuals would influence their propensity to be treated.

## Discussion

The goal of this study was to understand factors influencing decisions to be tested for COVID-19, and to examine how these considerations vary across social, economic, and psychological conditions. We find that a strong majority of people would consider their own and others’ vulnerability to COVID-19 in making decisions about testing, including the severity of their symptoms, proximity to someone infected, and the risk of infecting others. Resource considerations, including ability to pay for treatment, ability to work if positive, and the hospital’s capacity to treat them, are also important considerations for a minority of Indiana residents.

Though less commonly reported, resource motivations are socially stratified such that historically marginalized groups are disproportionately likely to consider these factors in their decisions. In particular, Black and Latino respondents were significantly more likely to cite resource constraints as a consideration in testing decisions, even after controlling for being uninsured and experiencing material hardship and general financial strain. Consistent with prior research, the basic survival needs of individuals and families often outweigh other considerations in healthcare decision-making, including prosocial motives like avoiding transmission to others [16, 17].

This pattern of findings has critical implications for population health and health disparities. Specifically, even brief delays in testing can have dire consequences for the efficacy of contact tracing strategies in transmission reduction [1]. If people in racial and ethnic minority groups and those in the lower socioeconomic strata avoid or delay testing due to inadequate resources, public health programs will be less effective in these populations. Given observed patterns of segregation and homophily in the US, this may contribute to higher transmission rates and the disproportionate burden of COVID-19 infection, hospitalization, and mortality in historically marginalized groups [18–20].

Additionally, our results are broadly supportive of research on strategic ignorance in the context of diagnostic testing and preventative medicine [5, 21, 22]. A growing literature on strategic ignorance suggests that people often ignore medical diagnoses or refuse testing despite being at risk [21, 22]. Reporting that testing decisions would be based on ability to pay for treatment or the capacity of hospitals to provide treatment may reflect instrumental futility, wherein knowing about being COVID-19 positive does not improve one’s ability to achieve a more positive outcome. Likewise, those who would consider their ability to continue working if they tested positive may avoid testing because it would produce a conflict between their personal financial security and the prosocial benefit of protecting others from contracting the disease. For those in precarious economic conditions, knowing about COVID-19 positivity could threaten their ability to provide for the basic material needs of themselves and their families. Finally, among those who have an immediate vulnerability to COVID-19 because they have been exposed, are experiencing severe symptoms, or might infect loved ones, having certainty about COVID-19 status, one way or the other, may reduce psychological distress. Under these circumstances, the need to know and the fear of adverse outcomes may outweigh the psychological comfort of remaining ignorant.

### Implications for public health policy and practice

Absent readily accessible, compulsory testing strategies, factors that influence decisions to get tested will likely lead to disproportionate testing in specific groups. People who report more worry about COVID-19 are more apt to seek testing. These people are more likely to already be taking protective measures, potentially resulting in routine underestimation of incident COVID-19 cases [23–25]. Testing strategies that target representative samples help with estimating the impact of this bias on existing case counts and shape which populations would benefit most from interventions to reduce COVID-19 transmission. Similarly, resource constraints more strongly influence testing decisions among racial and ethnic minorities and those who report financial strain. This group is less likely to have the resources to engage in COVID-19 protective behaviors. Policies targeted to alleviate financial strain, minimally including paid time off from work if sick or awaiting test results, may have effects on testing behavior as well as widespread social benefits [26, 27].

Coupled with these policies, our findings suggest that an opportunity exists to employ message framing and health literacy campaigns that appeal to people’s concerns about their own and others’ COVID-19 vulnerability. Recent studies suggest that worrying about COVID-19 is a strong predictor of risk perception and engagement in protective behaviors [24, 25]. Emphasizing the prosocial benefits of testing within an identifiable frame is likely to be more effective than presenting general statistics or self-interested appeals [28, 29]. At the same time, gaps in COVID-19 health literacy that have been fueled by false, contradictory, or complex information can be addressed by evoking social norms, patriotism, and collectivism [25, 30]. To make testing messaging more appealing to those whose concerns around COVID-19 are primarily economic, content might highlight the risk of income shocks due to untreated COVID-19, business closures precipitated by outbreaks, or risk of transmitting to other economically vulnerable friends and family members [31]. Any messaging will need to be culturally-specific given the wide range of concerns and beliefs identified here and in other research around testing, protective behaviors, and vaccination [28, 31–34].

### Limitations

While our study benefits from using a representative sample of Indiana residents, the sample provides insufficient power to assess testing intentions for some race/ethnic minorities in the United States who may either exhibit dynamic hesitancies or access issues, including American Indian and Alaska Native people and Asian or other Pacific Islander people. Similarly, Indiana is not representative of the entire US and the findings may not be generalizable to predominantly non-White populations in other parts of the country. Finally, the timing of the data collection allows us to highlight inequalities in testing motivations as mandatory testing had not started, but this also limits our ability to assess the effectiveness of policies and interventions to increase testing. Future studies may better address this question.

### Conclusions

The most important finding to emerge from this study is the potential for economic inequalities to exacerbate health disparities. The COVID-19 virus has disproportionately affected historically marginalized populations in the US, with the highest rates of infection, hospitalization, and mortality in racial and ethnic minority and lower-SES groups [18–20]. In parallel, public health measures to reduce COVID-19 transmission have led to devastating economic consequences that are also unequal in their impact. Estimates of unemployment and income shocks due to the partial closings of the economy have been most pronounced in Black and Latino communities, and among the less-educated and those living at or near the poverty line [14, 35, 36]. Likewise, we find that social determinants of health–such as race and ethnicity, financial insecurity, and access to health care–shape variation in testing motivation and intention, which may accelerate transmission in these populations and impede public health interventions. In short, social, health, and economic stratification processes are mutually reinforcing in the current pandemic.

## Supporting information

S1 File (DOCX)
