# Supplementary material for: If you build it, will they come? Social, economic, and psychological determinants of COVID-19 testing decisions
Source: PLoS One. 2021 Jul 14;16(7):e0252658. doi: 10.1371/journal.pone.0252658 (PMC8279331; doi:10.1371/journal.pone.0252658)
Supplement: S1 File — (DOCX) [file pone.0252658.s001.docx]

**Appendix S1. Additional information on sampling and weighting**

This research uses data drawn from the P2P – a stratified probability sample of households in Indiana, USA with an oversample of economically depressed, rural counties. Sampling, recruitment, and survey methodology were developed in collaboration with the National Opinion Research Center (NORC), and match the gold standard General Social Survey. The P2P uses the NORC National Sampling Frame to achieve an equal-probability multi-stage cluster sample of housing units for the state of Indiana. First-stage units were composed of multiple counties, with the largest urban areas selected to guarantee representation (e.g., the greater Indianapolis metropolitan area). Second-stage units were comprised of a block, group of blocks, or a census tract. At both stages, units were chosen with probabilities proportional to number of housing units. In the third stage, housing units were chosen with equal probability.

Data were collected face-to-face in respondents’ homes by professional interviewers employed by the Indiana University Center for Survey Research. Respondents were paid up to $120 for participation. The P2P was in the field from October 2018 to March 2020, with 90% of observations collected between January 2019 and February 2020. A total of 1,677 individuals completed the P2P study (Wave 1), and 1,579 of these consented to be contacted for future studies.

All P2P respondents who consented to future contact were eligible for the COVID-19 rapid response follow-up (Wave 2). Data collection began on March 28th and was completed on May 31st, 2020, during the height of the first wave of the pandemic in Indiana. Eligible participants were contacted through postal mail and email to invite participation. They were then recruited and consented into the study by phone. Data were collected by trained interviewers at the IU Center for Survey Research using computer assisted telephone interviewing software. Participants received a $20 gift card for participating. A total of 1,026 eligible P2P respondents took part in the follow-up (response rate 69%). Most attrition was due to inability to reach a respondent by phone after multiple contact attempts and voice messages. Additional information about the study can be found at <https://precisionhealth.iu.edu/get-involved/person-to-person.html>.

Since Indiana is predominantly white, the P2P oversampled racial and ethnic minorities to provide more robust information about people from minority groups. To ensure that our analyses are representative of individuals across the state of Indiana, we applied post-stratification weights. We weighted respondents to match the proportion of people in Indiana within their given age group (ages 15-24, 25-34 35-44, 45-54, 55-64, and 65+), sex (male and female), and racial group (White, Black, Latino, and Other). For example, the sum of our weights for respondents who are Latino men aged 15-24 was identical to the proportion of Indiana residents that are Latino men aged 15-24. In univariate statistical analyses, these weights were multiplied by each observed value prior to aggregation. In regression analyses, the weights were treated as sample weights that alter the extent to which each data point influences the fitting criterion. Observations with smaller weight had less impact on the final parameter estimates than observations with larger weights. The demographic characteristics of the weighted P2P sample are similar to Indiana as a whole.

**Appendix S2. Exploratory factor analysis of COVID worry**


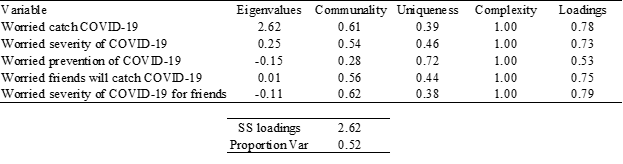


**Appendix S3. Regression results for agreement that criteria would affect decision to get tested for COVID-19 if symptomatic**


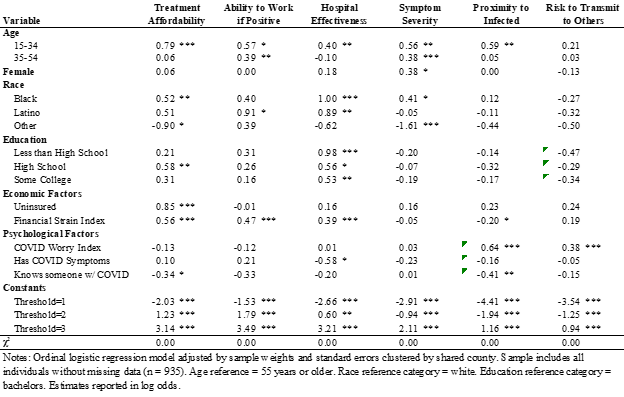


**Appendix S4. Graphical tests of the proportional odds assumption.**

The following graphs illustrate tests of the proportional odds assumption by examining whether slopes are parallel across values of each dependent variable. For each value of each independent variable, these figures illustrate values of the dependent variable (log-transformed) where the dependent variable = strongly agree minus values of the dependent variable (log-transformed) where the dependent variable = agree. When the differences across values of a given independent variable are not approximately identical, the proportional odds assumption does not hold well.

For example, the first figure below shows the association between each parameter value with treatment affordability (logged) where treatment affordability = strongly agree minus the association between each parameter value with treatment affordability (logged) where treatment affordability = agree. The difference in associations for Age 15-34 = No and Age 15-34 = Yes at values of treatment affordability = strongly agree and agree are roughly the same, suggesting that the proportional odds assumption holds for this variable. In contrast, the COVID worry index between the range of 0.750-1.000 has a somewhat smaller difference between values of treatment affordability = strongly agree and treatment affordability = agree than for values of the COVID worry index between the range of 0.000-0.750. This would suggest that the proportional odds assumption does not hold as well for the COVID worry index in estimating treatment affordability.

Overall, the proportional odds assumption holds quite well for most variables across most outcomes. High values of the COVID worry index may violate the proportional odds assumption for some outcomes; but relatively few respondents hold such high values on the COVID worry index.


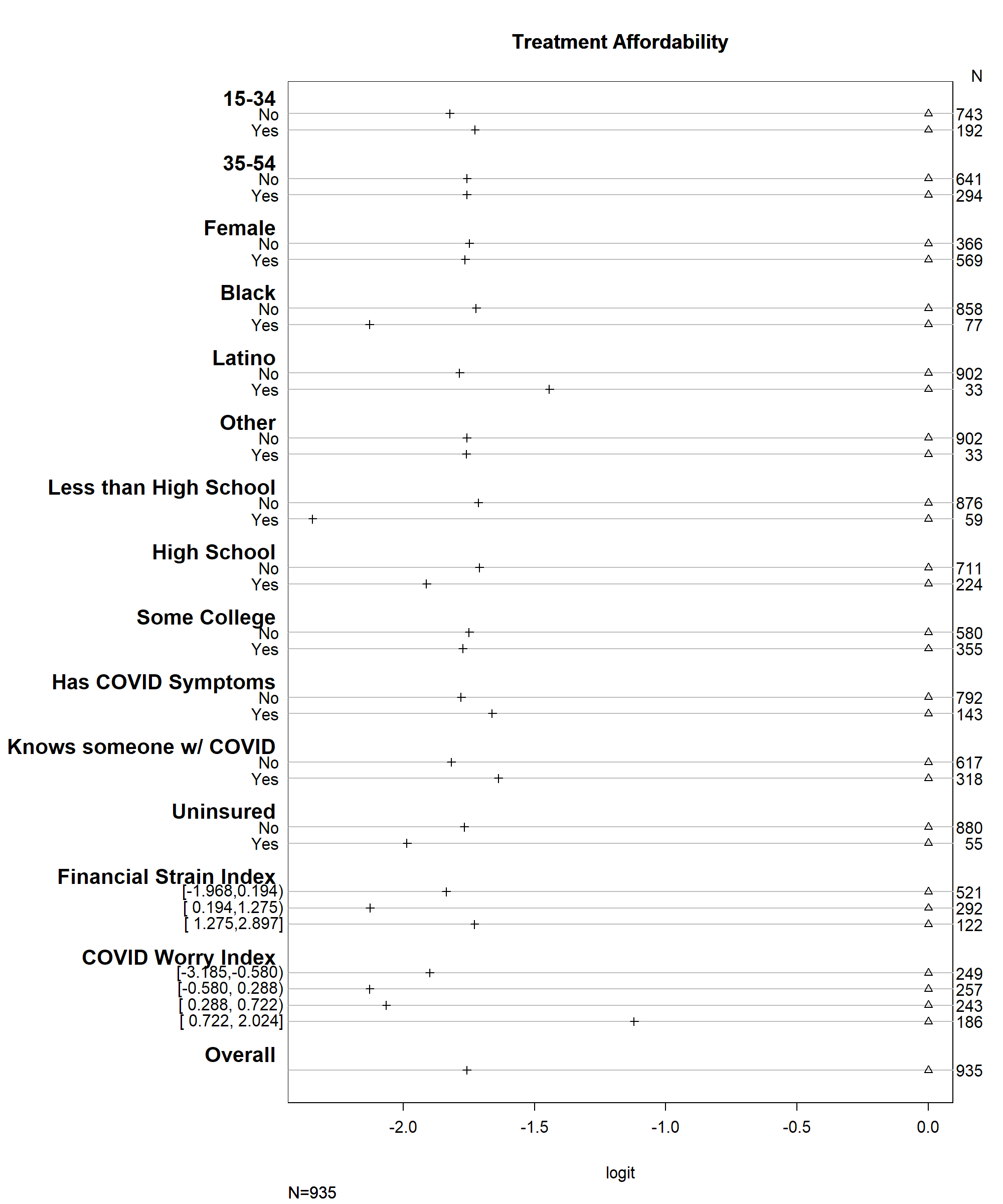

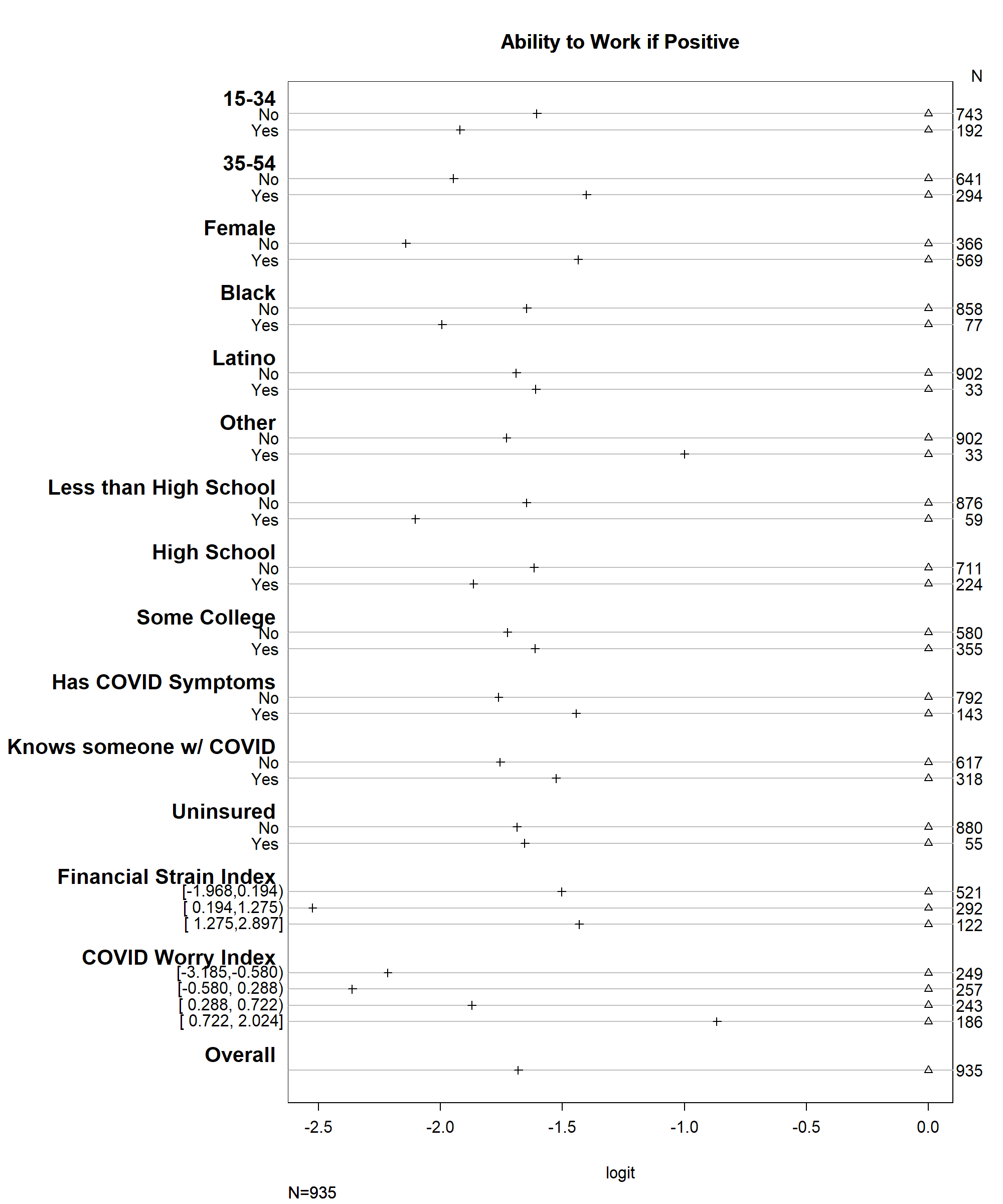

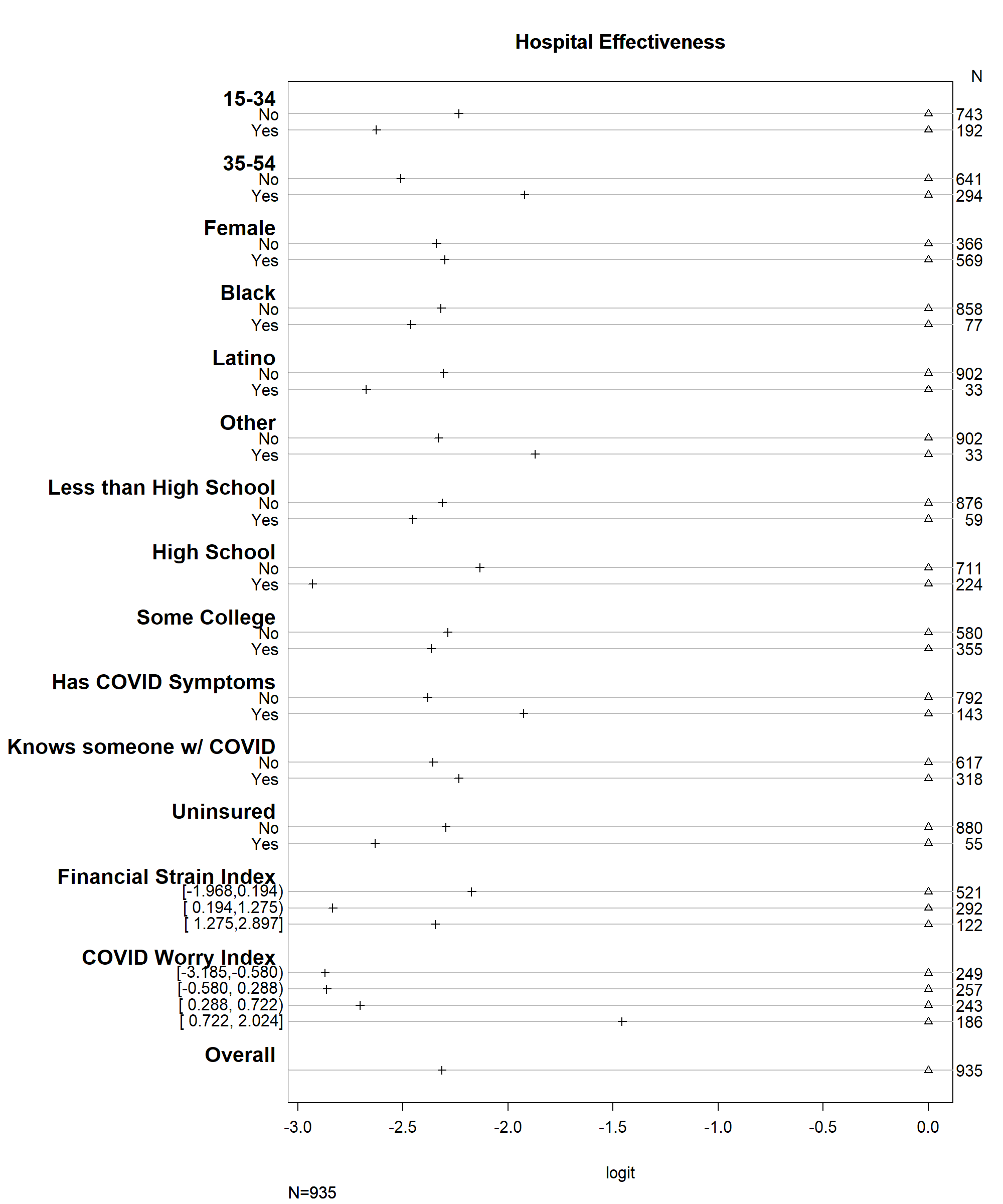

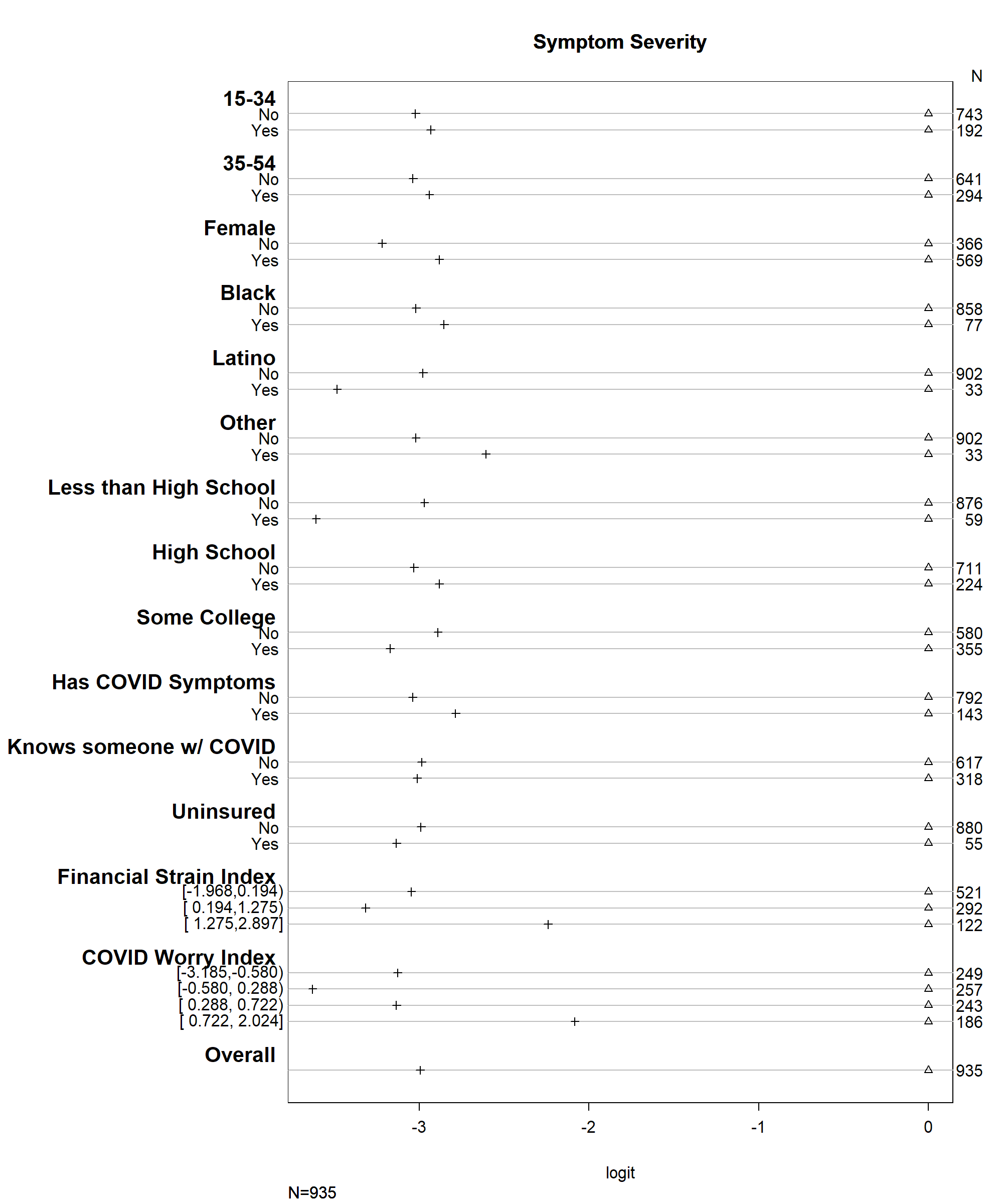

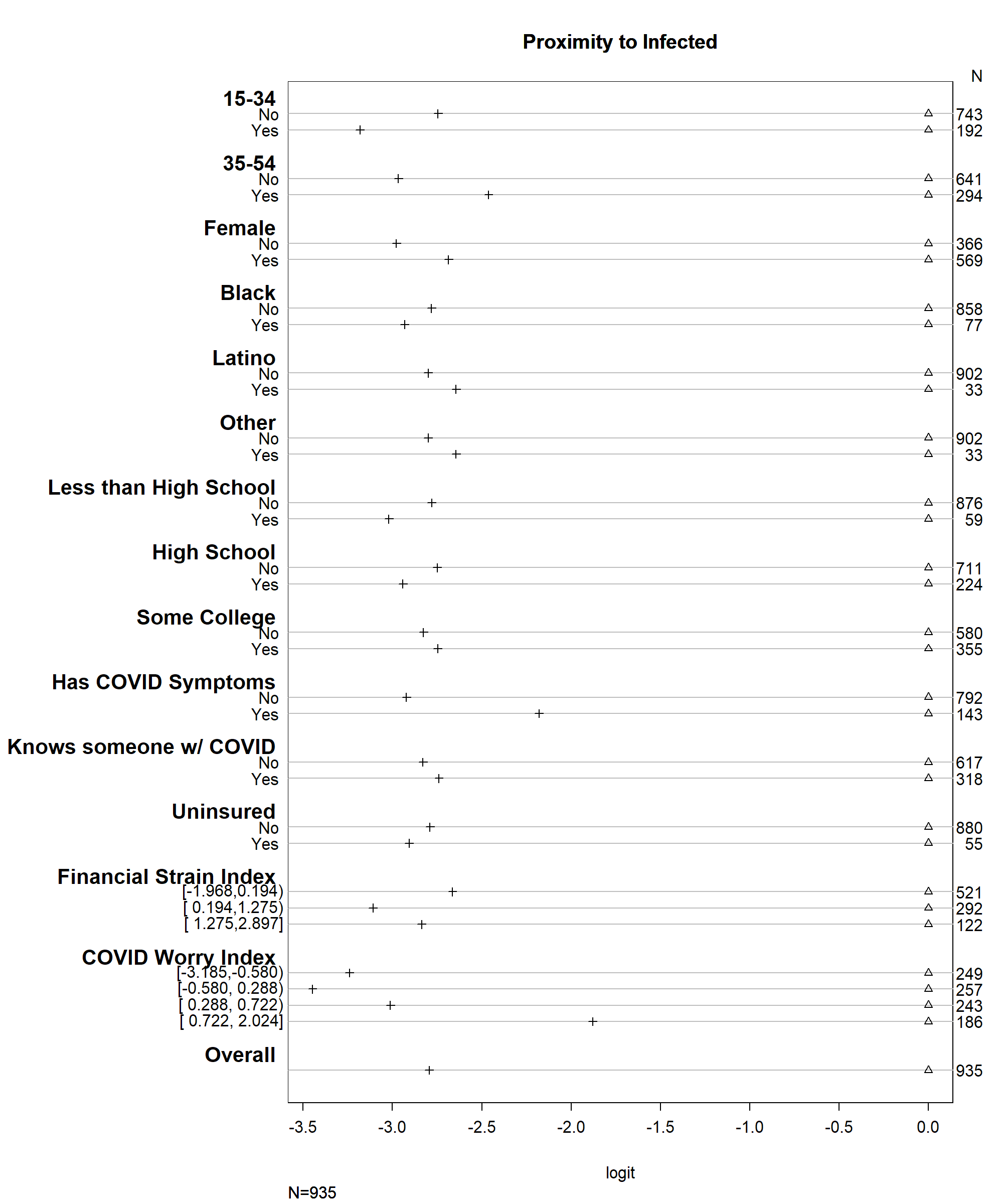


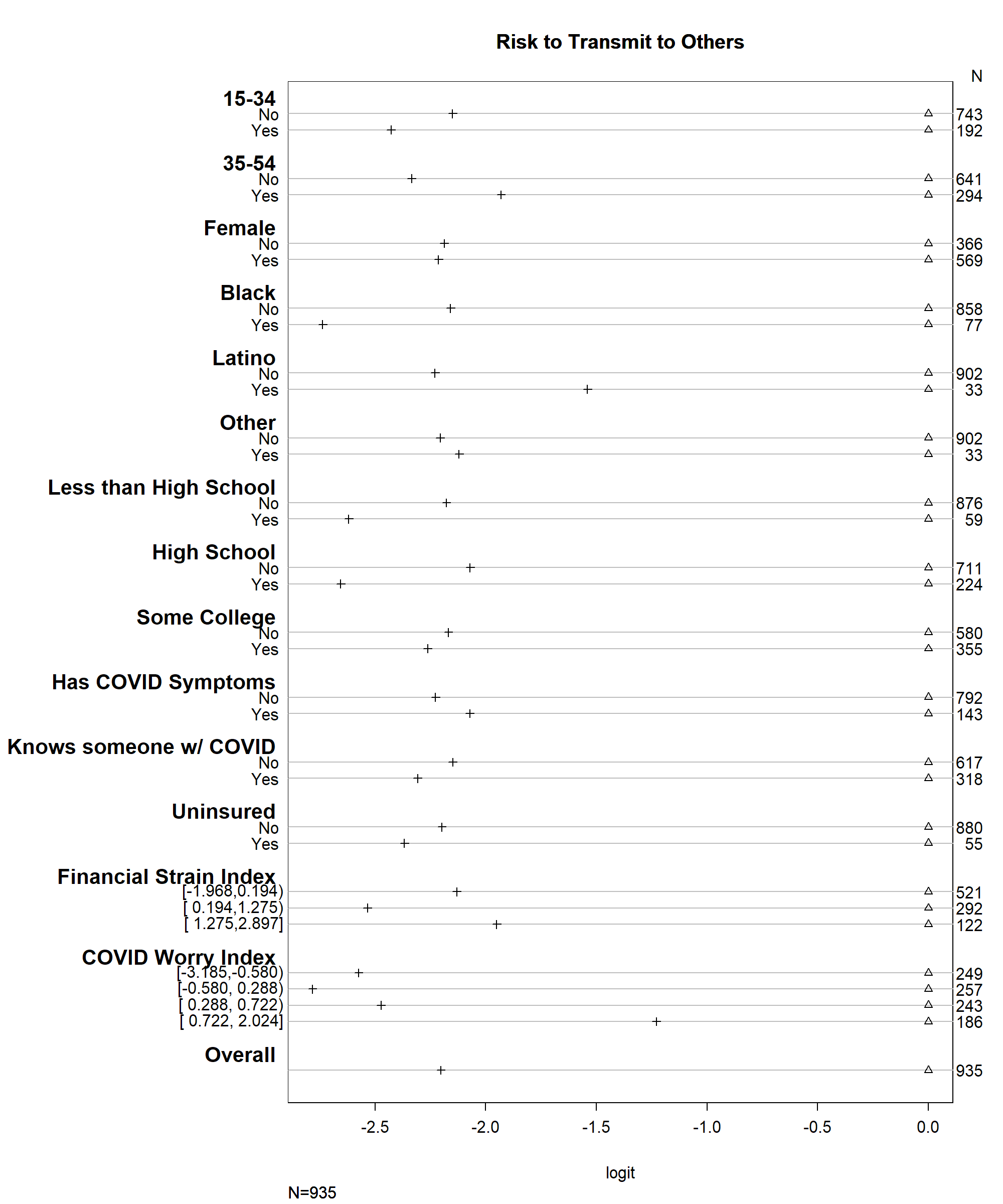


**Appendix S5. Factors associated with anticipated testing for COVID-19 by race/ethnicity, education, and age (with confidence intervals)**


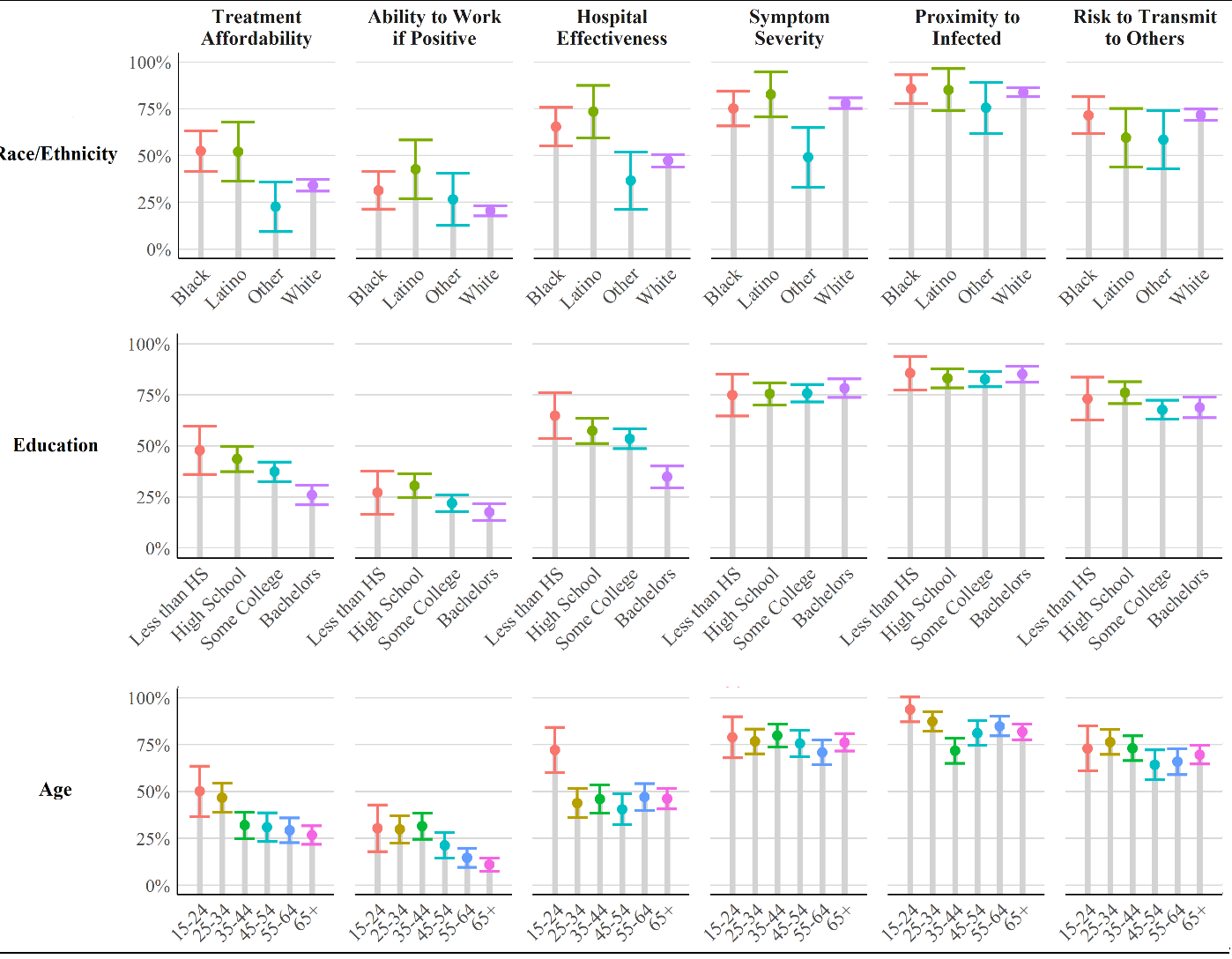


Note: Sample means estimated with survey weights. Sample includes all individuals without missing data (n = 935). All outcomes dichotomized into 1 = agreed or strongly agreed and 0 = disagreed or strongly disagreed. The bars are interpreted as the proportion of the demographic group category that endorses the respective testing motivation.
